# Forequarter amputation (upper limb and shoulder girdle) in a synovial sarcoma case–case report

**Published:** 2010-11-25

**Authors:** S Marinescu, C Giuglea, R Mihai, A Oporanu, S Manea, IP Florescu

**Affiliations:** Plastic and Reconstructive Microsurgery Clinic, ‘Bagdasar Arseni’ Clinical Emergency Hospital,BucharestRomania

**Keywords:** sarcomas, radical excision, forequarter amputation, immunohistochemical studies, shoulder girdle, metastasis

## Abstract

We are often confronted with severe cases–patients with very aggressive tumours that suppose a complex and in the same time radical approach–in our medical practice. The correct approach and management of such cases ensure both the surgical success and the patient survival. In this paper, we present the case of a young woman, who has been admitted in our clinic with a giant, irradiated tumour involving left axilla, shoulder and scapula. Due to the vast size of the tumour and to the fact that surgical biopsy revealed a poorly differentiated sarcoma; other clinics considered that the case above belongs to surgical therapy.  After the clinical examination, blood tests and diagnostic imaging, which allowed the correct evaluation of the case–tumour sizes and neighbouring tissue reports–we decided to perform tumour radical excision, respectively forequarter amputation, when the patient presented a satisfactory metabolic status. The presented case supports the idea that radical excision which might involve even mutilating amputations for extensive cancers can give patients a chance, even in desperate cases.

## Introduction

Synovial sarcomas represent 8–10% of all sarcomas and they most frequently affect adults in their decades from three to five. The median age of appearance is 31, 3 years old.  They are frequently misdiagnosed as benign tumours due to their small sizes and fine definition of their aspect[1,2]. They are the most frequently found sarcomas of upper limb, knee, hip and groin. They arise from mesenchymal pluripotent stem cells. Most of them are located in the periarticular regions, usually in close association with tendon sheats, bursae and joint capsules[3]. They are generally well circumscribed but in particular cases, can intermingle with muscles, tendons or can encapsulate neurovascular structures. Unlike other sarcomas, they can invade the neighbouring bone in 10–20% of the cases. Their most frequent metastastases are found in lungs, lymph nodes, liver or bone[4].

## Case report

We present the case of a patient, ML, aged 33, who was admitted in our clinic for an arm and axilla radionecrotic tumour. The tumour was first seen in the patient 9 months ago. It presented as a round mass, with 0.75 cm diameter. The tumour had an explosive growing in the past 3 months. She was investigated in an Orthopaedic Clinic, where the diagnostic imaging included MRI, and which revealed a heterogeneous tumour in close contact with the axilla vascular and nervous bundle, invading the rhomboids and deltoid.

The differential diagnosis included chondrosarcoma, liposarcoma, malignant fibrous histiocytoma, musculoskeletal tumors (percutaneous needle biopsy), osteochondroma and osteochondromatosis, osteosarcoma and synovial osteochondromatosis, etc.

Further, a tumour biopsy was performed–tissue fragments representing malignant tumour proliferation with small, round cells, having a reduced cytoplasm, hyperchromatic nuclei with a compact disposition and large areas of tumoral necrosis. These hystopathologic aspects are synonymous with a Ewing PNET tumour, but do not exclude a synovial sarcoma or a rabdomiosarcoma

Immunohistochemical studies revealed: EMA: positive in rare tumour cells; CK cocktail positive in tumour cells; S 100 negative in tumour cells; CD57 negative; CD 34 negative in tumour cells and positive in vessels; CLA negative in tumour cells and positive in small lymphocytes.  Thus, these tests are compatible with a low'grade synovial sarcoma diagnostic. 

The tumour had a substantial growth from the day of biopsy. Further, due to the tumour's large volume, surgical intervention was preceded by radiotherapy and chemotherapy (ifosfamide and doxorubicine). The tumour regressed a lot. When the patient was admitted in our clinic, she presented a 10/10 cm mass, involving the posterior left arm and axilla, fixed on the deep planes. The tumour was heterogeneous, ulcerated, with radiation necrosis, redness, oedema and tissue debris. 

**Figure 1:**
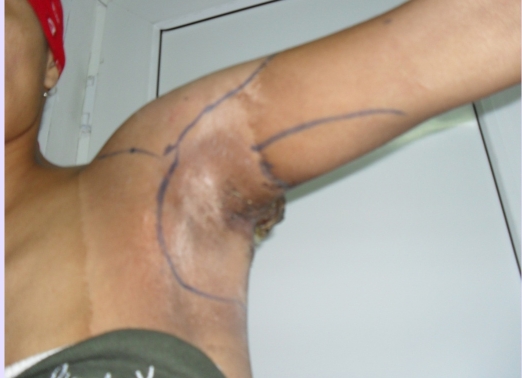
Preoperative aspect–anterior view

**Figure 2:**
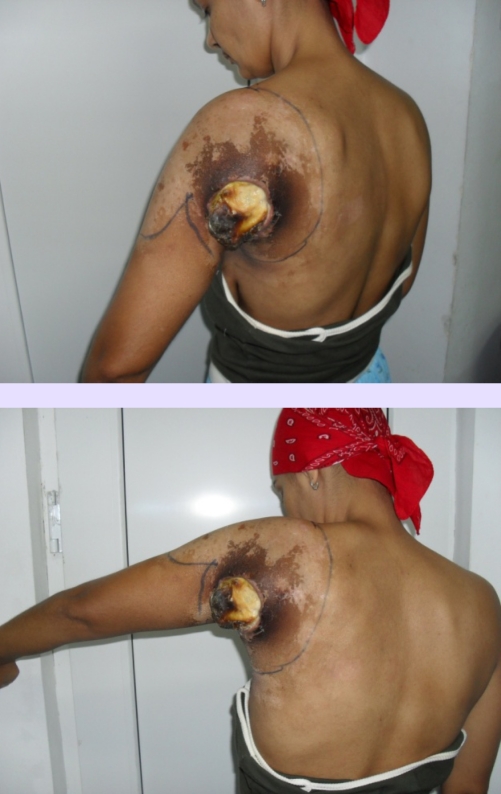
Preoperative aspect–posterior and lateral view

Diagnostic methods included; blood tests, ultrasonography and pulmonary radiology that revealed no metastases. 

Local infection (staphylococcus aureus and Raoultella (K) ornithinolytica) control was made possible by pre and postoperative administration of medaxone and gentamicyn.

**Radical surgical procedure** was imposed by: 1) the tumour's large dimension, 2) shoulder girdle muscle invasion, 3) close contact and invasion of axilla vascular and nervous bundle and 4) histopathological form–low–grade sarcoma–more aggressive and a higher rate of metastases[5,6,7,8].

Forequarter amputation was imposed as a life saving measure. Surgical intervention was carefully planned so as to allow long time cover of the amputation stump.We decided to perform an anterior approach, the patient being placed in a right lateral position.

**Figure 3:**
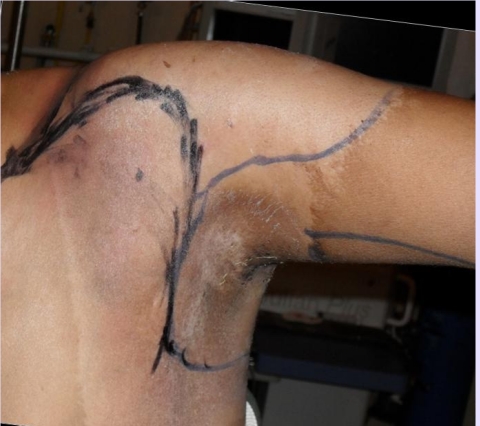
Preoperative marking follows the clavicle from lateral to sternocleidomastoidian muscle and goes to the deltopectoral groove, around the axilla.

**Figure 4:**
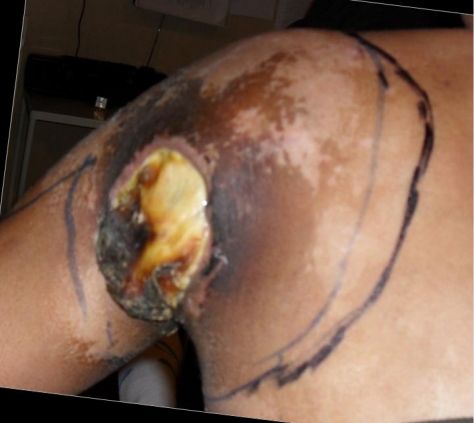
The marking goes posterior to the acromioclavicular joint and follows posterior and medial scapula vertebral border to its inferior angle.

The surgical technique consisted of an incision on the preoperative markings. These were deepened to the musculofascial layers. Platysma and supraclavicular nerves were cut. The clavicle was exposed and cut laterally to the sternocleidomastoidian with a Gigli saw. The tendons of pectoralis major, coracobrachialis and short head of the biceps were cut. Then, the subclavicular vessels were ligated.

**Figure 5:**
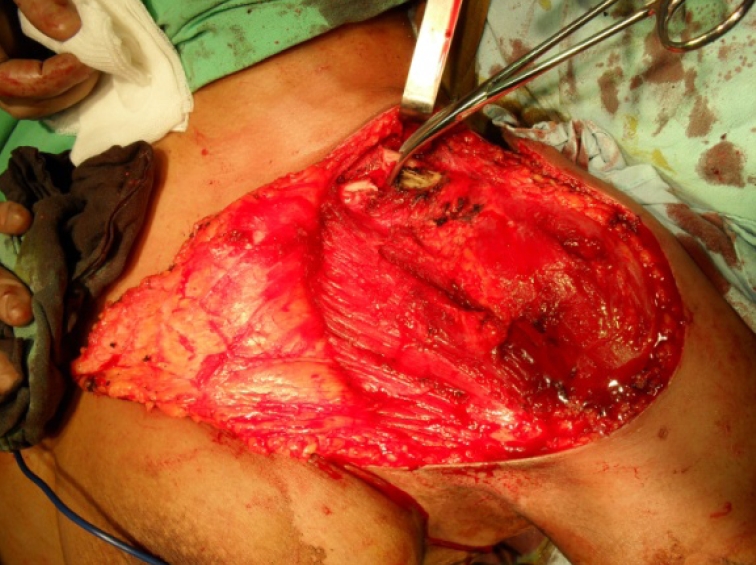
Intraoperative aspect

**Figure 6:**
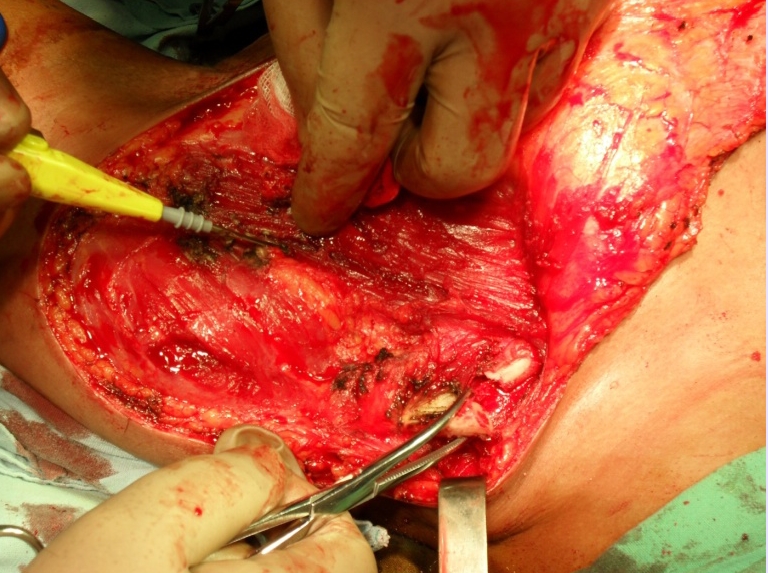
Intraoperative aspect–clavicle exposure and section, laterally to sternocleidomastoidian muscle

**Surgical technique:** After the subclavicular vessel ligature, brachial plexus branches were isolated, severed and let to retract. Scapula, trapezius, rhomboids, latissimus dorsi and seratus anterior were removed as the dissection progressed in the posterior plane [9].

**Figure 7:**
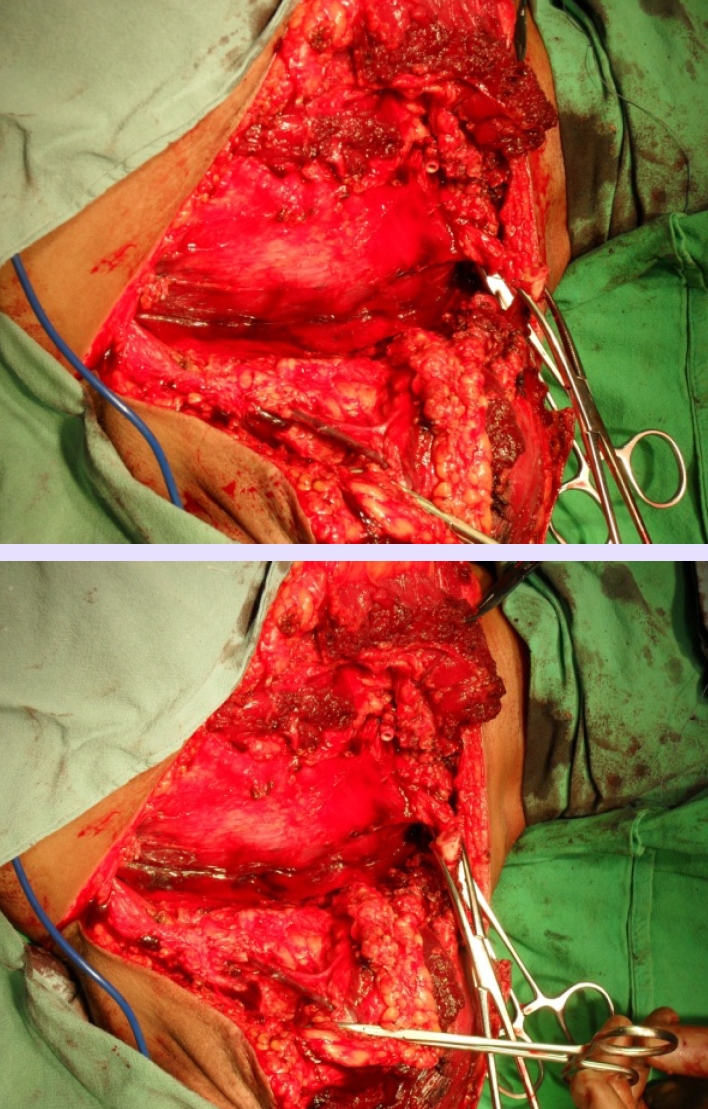
Intraoperative aspect–brachial plexus element section

**Figure 8:**
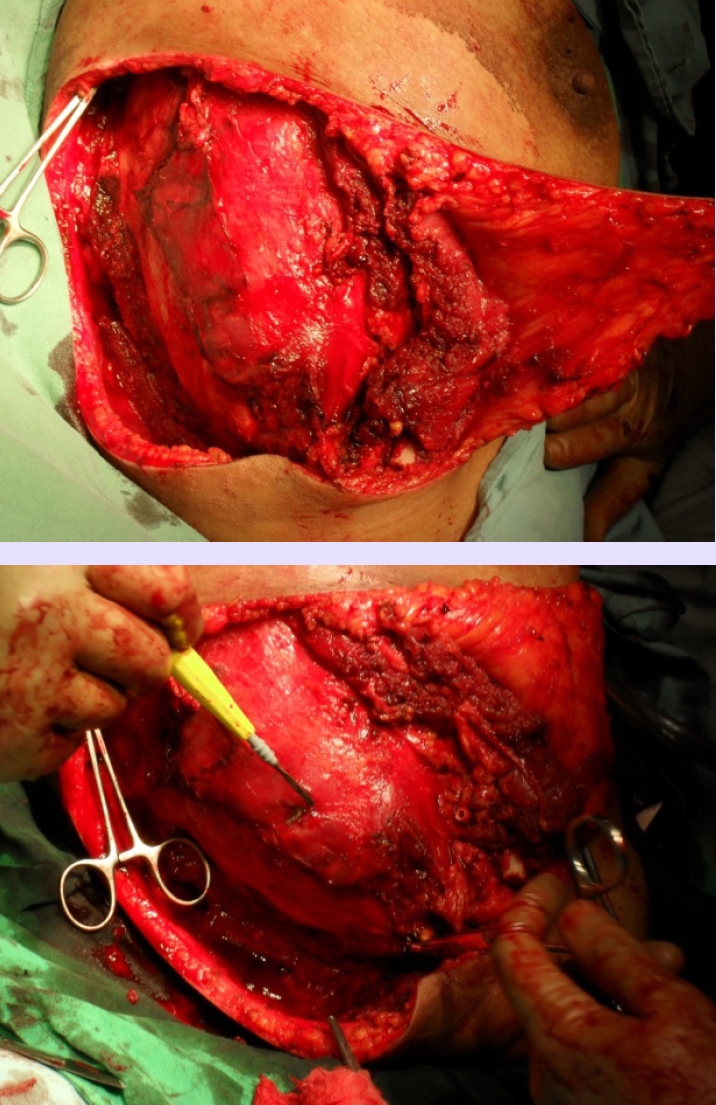
Intraoperative aspect–scapula and shoulder girdle muscle resection

**Figure 9:**
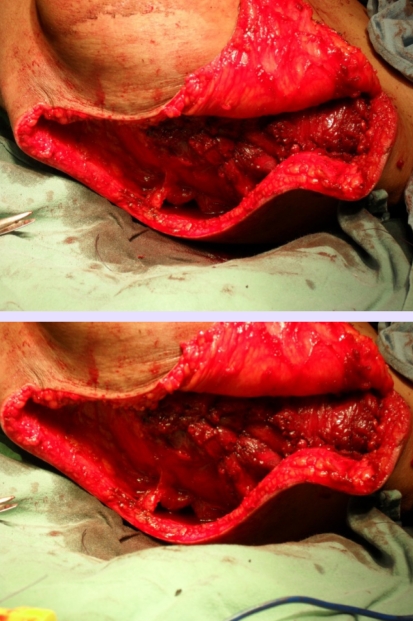
Intraoperative aspect fixing the skin flaps

**Figure 10:**
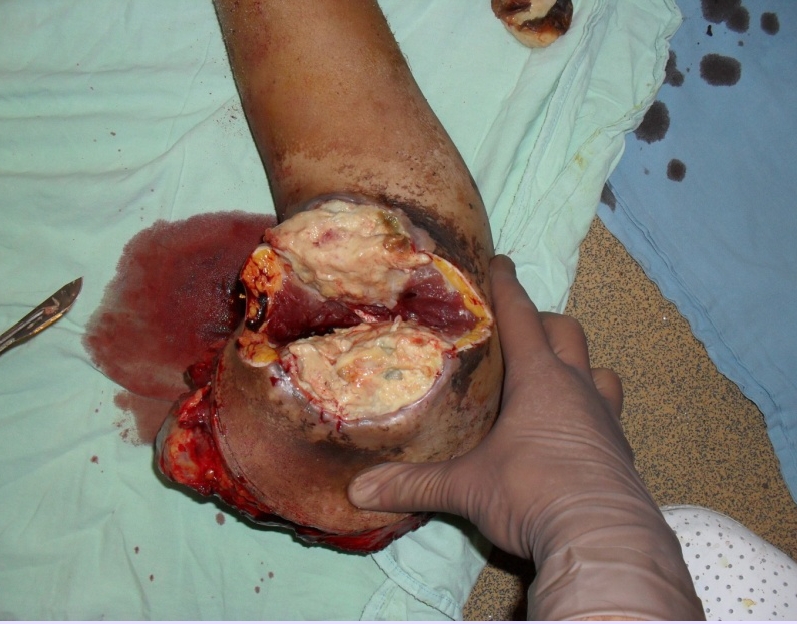
The amputated limb that included the tumour

**Figure 11:**
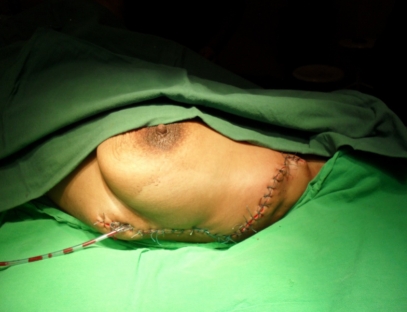
Final aspect final–skin suture

The histopathology revealed a sarcoma–like tumour proliferation, with small, round cells presenting radiation necrosis changes. Axillary lymph nodes presented degenerative changes with massive reactive sinus histiocytosis.

**Figure 12:**
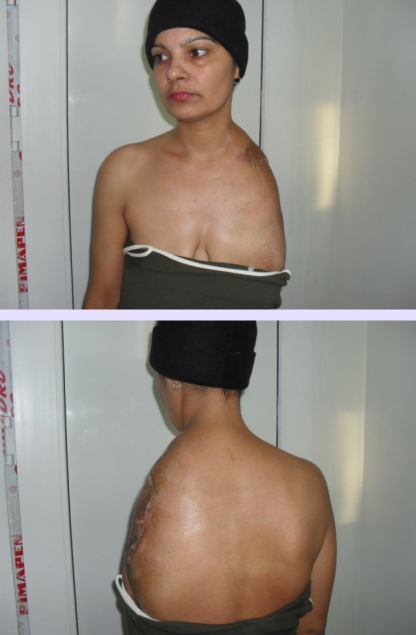
One–month postoperative aspect–anterior and posterior view

**Figure 13:**
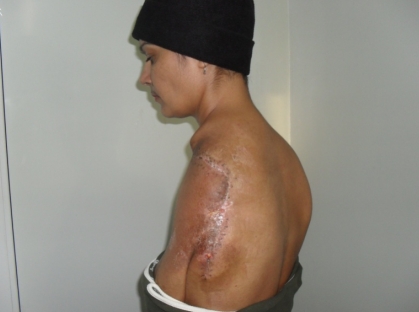
One–month postoperative aspect–lateral view

## Conclusions

- The ideal attitude would be tumour rapid diagnostic, establishing its gravity and, last but not least, immediate and correct therapeutic approach.
- By evaluating the tumour's extension both at surface and in depth, we shall be able to perform a radical excision.
- Synovial sarcomas need an aggressive therapeutic attitude. Whenever possible the approach would be a limb sparing surgery.
- The election treatment is a margin free disease large excision. This supposes, quite often, the neighbouring muscle resection or total amputation.
- In our case amputation was synonym with radical excision, being the only measure for a prognostic improvement.
- ‘Forequarter amputation’ represents a mutilating surgical intervention.
- Therapeutic option was imposed both as a need for radical excision and as a life saving measure.
